# Axonal regeneration after optic nerve crush in Nogo-A/B/C knockout mice

**Published:** 2008-02-04

**Authors:** Ying Su, Feng Wang, Shi-guang Zhao, Shang-ha Pan, Ping Liu, Yan Teng, Hao Cui

**Affiliations:** 1 Department of Ophthalmology, First Clinical College of Harbin Medical University, Harbin, China; 2 Department of Neurological Surgery, First Clinical College of Harbin Medical University, Harbin, China; 3 Key Laboratory for Cell Transplantation under the Ministry of Health, China

## Abstract

**Purpose:**

The axonal regeneration of retinal ganglion cells (RGCs) after optic nerve (ON) crush was investigated both in vivo and in vitro on Nogo-A/B/C knockout mice.

**Methods:**

The study used 20 Nogo-A/B/C knockout mice in the experimental group, and 20 C57BL/6 mice in the control group. Partial ON injury was induced by using a specially designed ON clip to pinch the ON 1 mm behind the mouse eyeball with 40 g pressure for 9 s. The left ON was injured in both groups, but the right ON was left untouched in the control group. Nogo-A/B/C mRNA was studied by in situ hybridization in both groups. GAP-43 was studied by immunofluorescence staining on frozen sections. RGCs were purified and cultured in DMEM medium containing B-27. Cells were then immunostained with both Thy1.1 and GAP-43 antibodies. The axonal growth of RGCs was calculated by a computerized image analyzer.

**Results:**

GAP-43 expression was significantly higher in the experimental group than in the control group (p<0.01). GAP-43 antibody binding was demonstrated in the axons of cultured RGCs. Axonal growth was significantly more active at every observed time point in the experimental group than in the control group (F=43.25, 32.16; p<0.01).

**Conclusions:**

Nogo genes play an inhibitive role in the axonal regeneration after ON injury, while Nogo-knockout is an effective way to eliminate this inhibition and accelerate axonal regeneration.

## Introduction

Glaucoma and optic nerve (ON) trauma may lead to optic nerve injury. It is widely known that central nervous system (CNS) neurons fail to regenerate after injury. This question has attracted intense investigation at both the preclinical and clinical levels. Regeneration failure has been attributed in part to proteins associated with CNS myelin and the scar that forms at an injury site. Furthermore, an ON trauma model can also be used as experimental research model to study CNS injury.

Nogo-A, Nogo-B, and Nogo-C are major protein species in oligodendrocytes and expressed in ON [1]. Among them, Nogo-A is the principal protein that prevents axonal outgrowth. A 66-amino loop structure located between two transmembrane domains named Nogo-66, which is common to all Nogo forms, is the main contributor to this inhibitory effect. Nogo-66 is expressed on the surface of oligodendrocytes [2] and functions by binding to an axonal Nogo-66 receptor (NgR) [3]. Nogo-66 is somewhat “fastidious” as regards to which environment it will express, as it is expressed in the myelin of a sciatic nerve transplant to the CNS but not in those to the peripheral nervous system (PNS). This feature, together with its in vitro activities, has confirmed Nogo-66 as a myelin-derived inhibitor to the axonal regeneration after injury.

Kim et al. [4] found that corticospinal axons of young adult Nogo-A knockout mice sprout extensively rostral to a transection after spinal cord injury. In this study we investigated axonal regeneration after optic nerve injury in young adult Nogo-A/B/C knockout mice.

## Methods

### Reagents

Sodium pentobarbital, paraformaldehyde (Shanghai Biologic Company, Shanghai, China), Vectashield mounting medium (H-1000, Vector Laboratories, Burlington, Ontario, Canada), GAP-43 antibody (Serotec, Raleigh, NC), DMEM medium and B-27 medium (Gibco, Burlington, CA), Hanks solution (Hyclone, Logan, UT), papain (Worthington, Lakewood, New Jersey), poly-lysine, bovine serum albumin, DNAase, Thy1.1 antibody, phosphate buffer (Sigma, St. Louis, MO), 5% goat serum (Zhongshan Company, Beijing, China), SABC (Boster Company, Wuhan, China).

### Equipment

Equipment included: surgical microscope (OMS-110, Topcon, Tokyo, Japan), 40 g power ON forceps (Martins Instruments, Tullingen, Germany, donated by Professor Gu Zhao-bin, Gifu University of Japan), anatomical microscope (SZ-PT, Olympus, Tokyo, Japan), fluorescence microscope (IX70, Olympus), optic microscope (Olympus), CO_2_ incubator (BB16HF, Heal Force, Hong Kong, China), ultraclean work table (D8C-010; Heal Force), incubation plate (Coster Company, Cambridge, MA).

### Generation and maintenance of Nogo-A/B/C knockout mutant mice

A Nogo-targeted embryonic stem cell clone was identified from the Omnibank sequence Tag database. Omnibank mutations were created, using insertional mutagenesis based on retroviral-based gene trap methodology. Heterozygotic mice harboring the disrupted allele were bred to C57Bl/6 mice for expansion in our animal housing facility and intercrossed to maintain the Nogo-A/B/C mutation on a hybrid 129SvEvBrd_C57Bl/6 background. The heterozygotic mice (9 or 12-week-old) were donated by Professor Xiulan Xu. The mice were raised in the laminar air flow cage rack (Suzhou Experimental Equipment Company, Suzhou, China). In the present study, animals were backcrossed to C57Bl/6 for three to six generations, and 7–14-week-old Nogo-A/B/C^−/−/-^ littermates were used as controls for all experiments. The present investigation adhered to the principles regarding the care and use of animals according to the American Physiologic Society, the Society for Neuroscience, and the ARVO Statement for the use of animals in ophthalmic and vision research.

### Animal grouping

The present study used 20 Nogo-A/B/C knockout mice (8 or 12-week-old) in the experimental group, and 20 C 57BL/6 control mice(8 or 12-week-old) in the control group. To depict the axonal growth at successive time points, both groups were further divided into three subgroups representing the first day (n=7), third day (n=7), and seventh day (n=7) outcomes, respectively.

### Surgical procedure

Prior to ON crush, general anesthesia was induced in each animal with an intraperitoneal injection of 1% sodium pentobarbital solution (50 mg/kg bodyweight). A surgical microscope was used to visualize the ON, which was exposed through a superior temporal approach. A 1–1.5 cm incision was made in the skin above the right orbit. The dural sheath of the ON was opened longitudinally, from which the ON was then gently detached before it was crushed at 1 mm distal to the eyeball by an ON forceps with 40 g pressure for 9 s. Injury to the ophthalmic artery was carefully avoided during the procedure. Nerve injury was verified by the appearance of a clearing at the crush site; the vascular integrity of the retina was verified by funduscopic examination after dilating the pupil with atropine. The left ON was crushed in both groups, but the right ON was left untouched in the control group.

### Preparation for pathological and immunohistochemistry examinations

Seven days after ON crush, thoracotomy was performed. The animals were perfused through the heart with heparin saline followed by 4% paraformaldehyde, including diethypyrocarbonate (DEPC) for in situ hybridization. Eyes with ON segments up to the optic chiasm were postfixed overnight, and transferred to a 30% sucrose solution overnight (4 °C) and including DEPC for in situ hybridization overnight (4 °C). Frozen sections (15 μm) were cut longitudinally on a cryostat, thaw mounted onto coated glass slides, and stored at −80 °C until further use.

Digoxygenin-labeled sense and antisense RNA probes were generated as described [5]. The serial number (NM-57142) and cDNA array of *Nogo* gene was achieved from GenBank. The probe was designed with Primer 3 software (Whitehead Institute for Biomedical Research, Cambridge, MA). The common probe, recognizing all three isoforms of Nogo, contains transcript A sequence between nucleotides 2535 and 4678.

Cryostat sections (15 μm) were mounted on Superfrost-Plus slides. Sections were post-fixed in 4% paraformaldehyde and PBS, acetylated in 0.1 M triethanolamine and 0.25% acetic anhydride, and permeabilized for 20 min in 1% Triton X-100 and PBS. Hybridization was performed overnight in 5x300 mM NaCl, 30 mM sodium citrate, pH 7.0 (SSC) buffer containing 50% formamide and 2% blocking reagent at 68 °C. Two rigorous washes were performed in 0.2X SSC at the same temperature for 1 h, and signals were detected with alkaline phosphatase-coupled anti-digoxigenin antibodies using spreptavidin-biotin-peroxidase complex (SABC) as color reaction substrates.

### Immunofluorescence staining

GAP-43 monoclonal antibody (1:500 dilution) was used in all cases. Secondary antibodies conjugated to distinct fluorescence (1:500 fluorescein-conjugated anti-sheep IgG) were used, and coverslipped with antifade medium.

### Isolation and culture of retinal ganglion cells

The eyes of the mice were collected after they were given a lethal dose of sodium pentobarbital. Retinas were isolated and dissected under an anatomic microscope to remove visible vessels before they were washed three times in Hank's balanced salt solution.

RGCs from the retina were isolated and purified as previously described [6,7]. Briefly, the retinal tissue was dissociated into single cells in Eagle's minimum essential medium that contained 15 U/ml papain and 70 U/ml collagenase. The cell suspension was incubated successively in a polypropylene tube coated with a monoclonal anti-rat macrophage IgG to remove macrophages, and then in another one coated with a monoclonal anti-rat Thy 1.1 IgG. Finally, the tube was gently washed five times with PBS. RGCs were collected by centrifugation at 700xg for 5 min and then cultured at a concentration of 6x10^6^ cells/ml in DMEM that contained 100 U/ml penicillin, 100 μg/ml gentamicin, and B-27. Cells were then seeded into poly-lysine coated 24-well plates and incubated at 37 °C with 5% CO_2_ ventilation. Half of the medium was renewed with cytarbine (20 μg/ml) added at certain intervals. Cells were examined daily under phase-contrast microscope.

### Immunocytochemistry

RGCs were fixed in 4% paraformaldehyde for 15 min and blocked with 5% goat serum. After three washes with PBS, the cells were incubated successively with primary antibody anti-GAP-43 (1:1000) at 4 °C for 24 h, secondary antibody goat-anti-rabbit IgG at 37 °C for 1 h, and SABC at 37 °C for 30 min. Between each step, cells were washed three times with PBS. After coloration with DAB, dehydration, and dimethyl benzene treatment, slides were mounted and examined with phase-contrast microscopy.

### Statistical analysis

The data were analyzed by the two-tailed Student *t*-test, using software (Origin ver. 6.0, OriginLab Corp., Northampton, MA).

## Results

**Figure 1:**
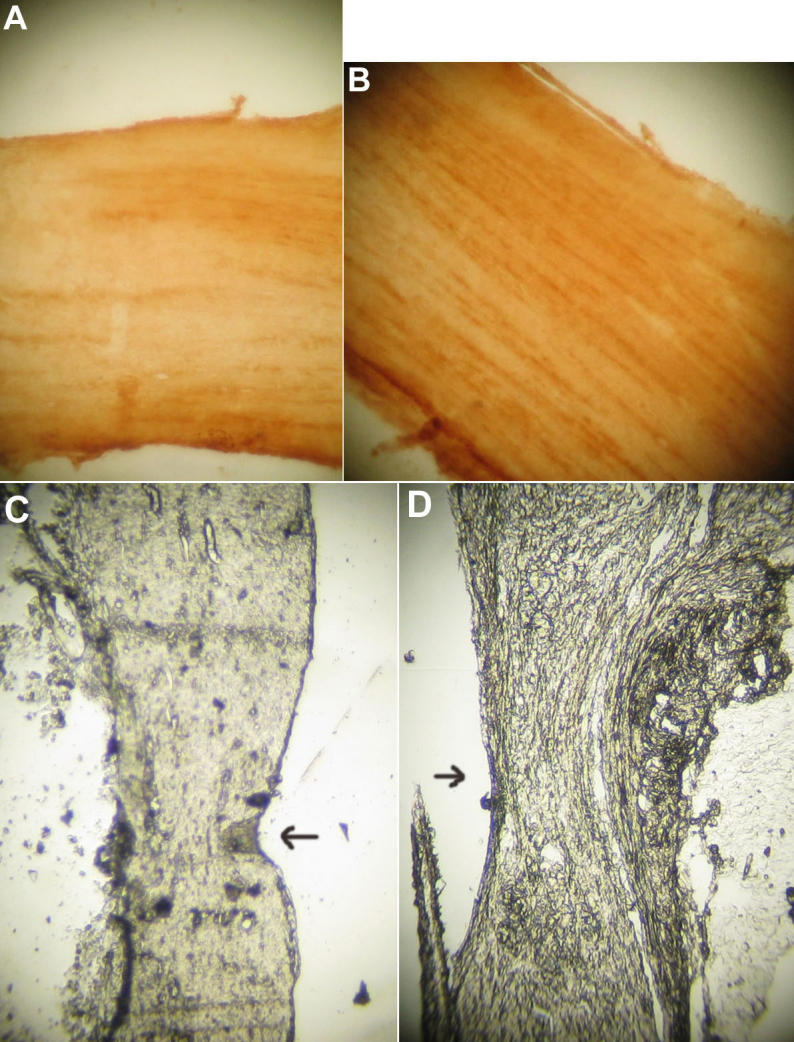
Expression of Nogo-A/B/C mRNA in optic nerve. Expression of Nogo-A/B/C mRNA in optic nerve of experimental group (Nogo-A/B/C knockout mice) and control group (C57BL/6 mice) as visualized by in situ hybridization stained as yellow. Positvie area of stain of Nogo-A/B/C expression of experimental group was 1.36±0.05x10^2^ μm^2^ whereas that of control group was 26.56±1.25x10^2^ μm^2^. There was significant difference positvie area of stain of Nogo-A/B/C expression between experimental group and control group(p<0.01). **A**: Little or no expression of Nogo-A/B/C mRNA was observed in the ON of Nogo-A/B/C-knockout mice. **B**: Expression of Nogo-A/B/C mRNA was demonstrated in the ON of young adult normal mice. The scale bars represents 20 μm. **C**: The crush position (arrow) of optic nerve of experimental group (Nogo-A/B/C knockout mice) can be investigated under normal light microscopy. The scale bars represent 20 μm. **D**: The crush position (arrow) of optic nerve of control group(C57BL/6 control mice) can be investigated under normal light microscopy. The scale bars represent 20 μm.

Expression of Nogo-A/B/C was in the cytoplasm of oligodendrocytes of optic nerve which arrange like a line of pearl. Stain of brown indicates positive expression of Nogo-A/B/C. Expression of Nogo-A/B/C was calculated using image analysis software. The images were taken using a digital camera mounted on a microscope. The analysis was performed using Image-Pro Plus version 4.0 (Media Cybernetics, Silver Spring, MD). Positvie area of stain of Nogo-A/B/C expression of experimental group was 1.36±0.05x10^2^μm/m^2^, whereas that of control group was 26.56±1.25x10^2^μm/m^2^. There was significant difference positvie area of stain of Nogo-A/B/C expression between experimental group and control group (p<0.01). Little or no expression of Nogo-A/B/C mRNA was observed in the ON of Nogo-A/B/C-knockout mice (Figure 1A). Expression of Nogo-A/B/C mRNA was demonstrated in the ON of young adult normal mice (Figure 1B).

**Figure 2:**
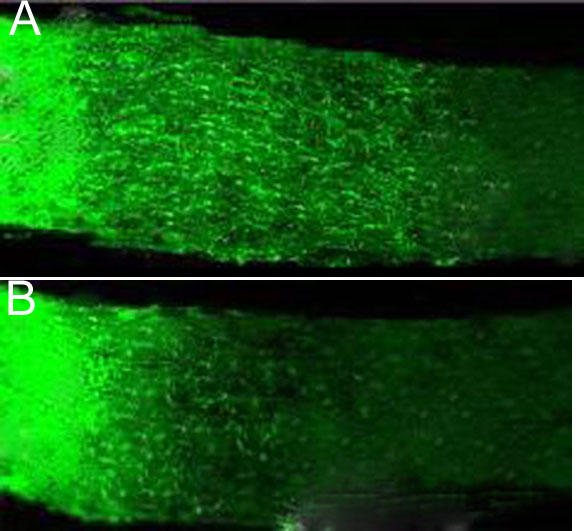
Expression of GAP-43 in experimental group (Nogo-A/B/C knockout mice) and control group (C57BL/6 mice; GAP-43 stain). **A**: There is more expression of GAP-43 in experimental group (green fluorescence stain). Regenerated axon can be investigated under immunofluorescence microscope. There was significant difference in stained positive areas on the ON sections between experimental group (Nogo-A/B/C knockout mice) and control groups (C57BL/6 control mice). **B**: There is less expression of GAP-43 in the control group (GAP-43 stain). The scale bars represent 20 μm.

GAP-43 is located in the regenerative axon of neurite. Expression of GAP-43 stained with green fluorescence in Figure 2A,B indicates regeneration of axon of optic nerve. Axon of positive GAP-43 expression distributes along the longitudinal axis of optic nerve. GAP-43 expression of optic nerve increased significantly with the survival time.

There was a significant difference in the size of the areas that stained positively for GAP-43 on the ON sections between experimental(Nogo-A/B/C knockout mice) and control groups (C57BL/6 control mice; p<0.01; Table 1). The positive area of GAP-43 stain of the experimental group increased significantly from one day to seven days after crush. There was little increase of the positive area of GAP-43 stain of the control group.

**Table 1:** Stained positive area of experimental and control groups (mean±SD).

**Time (day)**	**Positive area of stain of experimental group (10^2^µm^2^)**	**Positive area of stain of experimental group (10^2^µm^2^)**	**t**	**p**
1	5.06±0.26	0.36±0.05*	2.59	<0.01
3	16.28±1.29	0.52±0.09	3.12	<0.01
7	26.32±2.56**	0.88±0.25**	3.95	<0.01
	F	45.36	2.85	<0.01
	p	<0.01	<0.005	

There was significant difference in stained positive areas on the ON sections between experimental(Nogo-A/B/C knockout mice) and control groups (C57BL/6 control mice). The positive area of GAP-43 stain of the experimental group increased significantly from one day to seven days after crush. There was little increase of the positive area of GAP-43 stain of the control group. Comparison at first post-injury day, *p<0.01; comparison at seventh day, **p<0.01.(asterisk means positive area of stain of experimental group and control group at first post-injury day, double asterisk means positive area of stain of experimental group and control group at seventh day).

### In vitro axonal growth of the experimental and control groups

Adherence of RGCs began at 12 h after they were seeded into poly-lysine- coated 24-well plates and incubated at 37 °C 5% CO_2_ ventilation, and finished in 24 h. After this, a single layer of round and oval RGCs was seen on the plates.

**Figure 3:**
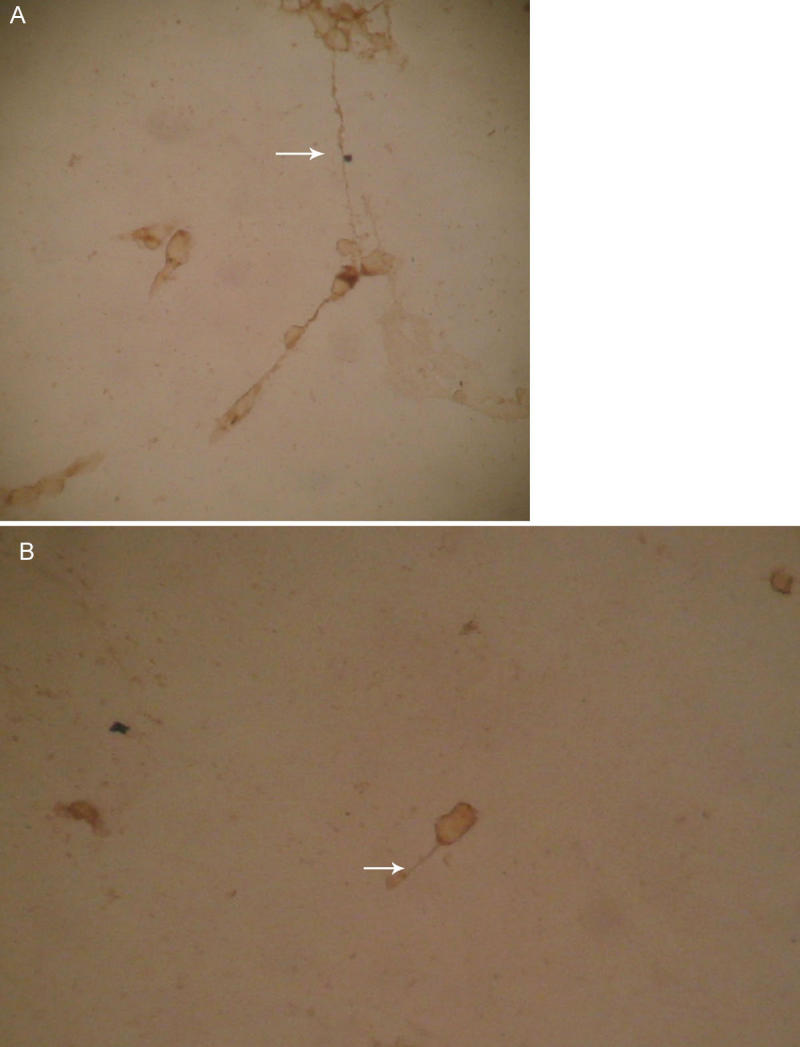
Axonal length of cultured RGCs. Axonal length of cultured RGCs of experimental group (Nogo-A/B/C knockout mice) and control group (C57BL/6 control mice) in the third day. The RGCs were purified and cultured as described above. The body of RGCs is round or oval. The RGCs were stained with GAP-43 antibody. The regenerative axon was stained as yellow. **A**: Axonal length was longer in the third day for the experimental group (Nogo-A/B/C knockout mice). **B**: Axonal length of the control group (C57BL/6 control mice) of the same day.(datas can be seen in Table 2). The scale bars represent 40 μm (GAP-43 stain).

We investigated axonal generation by staining of GAP-43. Axonal length was longer in the third day for the experimental group (Nogo-A/B/C knockout mice; Figure 3A) than that of the control group (C57BL/6 control mice) of the same day (Figure 3B).

Axonal length was calculated using image analysis software. The images were taken using a digital camera mounted on a microscope. For neurite length determinations, the neurites were traced and the morphometry analysis was performed using Image-Pro Plus version 4.0 (Media Cybernetics, Silver Spring, MD). The length of the axon was defined as the distance from the soma to the tip of the process. An average of 50 neurons from each group was selected from a number of chamber slides. The difference in axonal length of the experimental group (Nogo-A/B/C knockout mice) increased from the first day to the seventh day after crush. The axonal length of the control group (C57BL/6 control mice) increased from the first day to the third day, then decreased from the third day to the seventh day. The difference in axonal length between the experimental group (Nogo-A/B/C knockout mice) and the control group (C57BL/6 control mice) was not significant (p>0.05) on the first postoperative day. However, the difference in axonal length became obvious by the third and seventh days between the experimental and control groups. (p<0.01; Table 2).

**Table 2:** Axonal length of retinal ganglion cells of different time (μm mean±SD).

**Culture time (day)**	**Experimental group**	**Control group**	**t**	**p**
1	2.63±1.35	2.52±1.32	0.96	>0.05
3	23.39±5.56	9.32±2.24	3.26	<0.01
7	13.32±3.12	6.15±2.36	3.15	<0.01
	F	43.25	32.16	
	p	<0.01	<0.01	

The difference in axonal length between experimental group (Nogo-A/B/C knockout mice) increased from the first day to seventh day after crush. The axonal length of control group (C57BL/6 control mice) increased from the first day to third day, then descreased from the third day to seventh day. The difference in axonal length between experimental group (Nogo-A/B/C knockout mice) and control group (C57BL/6 control mice) was not significant (*p>0.05) on the first postoperative day. However, the difference in axonal length was significant by the third and seventh days between experimental group and control group.

## Discussion

Several myelin inhibitors of axon growth, including NogoA [2,8,9], myelin-associated glycoprotein [10], and oligodendrocyte-myelin glycoprotein [11], exert their effects through the Nogo receptor, and p75 [3].

The Nogo gene has been cloned and its product appears to be a new member of the reticulon family [12]. An extracellular segment composed of 66 amino acid residues between two transmembrane domains, known as Nogo-66, is the major functional domain of this protein [3].

A neutralizing monoclonal antibody, neurite outgrowth inhibitor 1 (IN-1), has recently been confirmed to target the N-terminal domain of Nogo-A encoded by exon 3 [13]. When IN-1 is applied to several CNS injury models, including spinal cord lesions and cortical lesions, there is notable neuroanatomical and behavioral recovery compared with controls [14]. These results suggest that the Nogo-NgR system is accountable for, to a large degree, the inhibitory activity that is responsible for the prevention of CNS axonal regeneration after injury.

Increased expression of Nogo-A in RGCs after ON injury indicates that Nogo-A may play an inhibitory role in axonal regeneration after ON injury.

The expressed GAP-43 is located in the growth cone of the regenerating axon. Positive expression of GAP-43 in our figures indicate regeneration of axon of optic neve.

There was a significant difference in the area of ON stained positive for GAP-43 between the experimental group(Nogo-A/B/C knockout mice) and the control group(C57BL/6 mice) in this study. Nogo is important inhibitor for regeneration after CNS injury. Knockout of Nogo-A/B/C gene can lead to regeneration after optic nerve injury in this study. The results indicate that Nogo-A/B/C plays an important role in the inhibition of axonal regeneration after ON injury.

The significant difference in the length of axons regenerated in vitro between the experimental and control groups on the third and seventh postoperative days suggests that Nogo-A/B/C elimination is effective in enhancing axonal regeneration of RGCs in at least in vitro experiments.

The analysis on mice with various degrees of gene disruption targeted at Nogo isoforms has been reported by three groups. Kim et al. [11] observed significant corticospinal tract fiber sprouting in young Nogo-A/B^−/−^ mice compared with wild-type and Nogo-A/B^+/−^ mice. Simonen et al. [15] noted moderate corticospinal tract fiber regeneration in Nogo-A^−/−^ mice. However, Zheng et al. [16] saw no significant regeneration in either Nogo-A/B^−/−^ line or Nogo-A/B/C^−/−^ line mice above that of wild type. It is believed that the varying degrees of regenerative capacity after Nogo elimination can be attributed to the differences in genetic backgrounds of the mice used in the experiments [17].

Increased expression of Nogo-A in RGCs after ON injury indicates that Nogo-A may play an inhibitory role in axonal regeneration after ON injury.

There was a significant difference in the area of ON stained positive for GAP-43 between the experimental group and the control group in this study. The expressed GAP-43 is located in the growth cone of the regenerating axon. The results indicate that Nogo-A/B/C plays an important role in the inhibition of axonal regeneration after ON injury.

The significant difference in the length of axons regenerated in vitro between the experimental and control groups on the third and seventh postoperative days suggests that Nogo-A/B/C elimination is effective in enhancing axonal regeneration of RGCs in at least in vitro experiments.

Several myelin inhibitors of axonal growth, including Nogo-66, myelin-associated glycoprotein, and oligodendrocyte-myelin glycoprotein, exert their effects through NgR. Further investigation of NgR may reveal whether NgR gene knockout enhances axonal regeneration after ON injury even more effectively.
